# Vermilionectomy for Actinic Cheilitis Treatment—Report of 03 Cases and Brief Literature Review

**DOI:** 10.1155/crid/9721944

**Published:** 2026-05-21

**Authors:** Bruno Teixeira Gonçalves Rodrigues, Eduardo Martins Falcão, Caroline Cavaignac Silva, Paula Granha Porto, Ana Carolina Silva Custódio, Allan Ribeiro Bastos, Verônica dos Santos Lopes Serrano, Henrique Martins da Silveira, Danilo Passeado Branco Ribeiro, Mônica Simões Israel

**Affiliations:** ^1^ Oral and Maxillofacial Surgery, Pedro Ernesto University Hospital, State University of Rio de Janeiro, Rio de Janeiro, Brazil, uerj.br; ^2^ Department of Oral Medicine, Faculdade São Leopoldo Mandic, Rio de Janeiro, Brazil, slmandic.edu.br; ^3^ Oral Medicine, Department of Diagnosis and Therapeutics, School of Dentistry, State University of Rio de Janeiro, Rio de Janeiro, Brazil, uerj.br

**Keywords:** management, oral medicine, oral potentially malignant disorder

## Abstract

Actinic cheilitis (AC) is a common potentially malignant disorder that primarily affects the lower lip due to chronic ultraviolet B exposure. Severe epithelial dysplasia in AC carries a significant risk of progression to squamous cell carcinoma, necessitating definitive treatment. Vermilionectomy is considered the treatment of choice in high‐risk lesions, offering both functional and aesthetic restoration. This study reports the clinical outcomes of three cases of AC with severe dysplasia treated via vermilionectomy. Three patients (two males, one female) with a histopathologically confirmed diagnosis of AC exhibiting severe epithelial dysplasia underwent vermilionectomy under local anesthesia. Surgical management involved excision of the affected vermilion to the muscular plane, hemostasis with electrocautery, and advancement of labial mucosa for reconstruction, followed by suture closure with 4‐0 nylon. Postoperative recovery, complications, and aesthetic outcomes were assessed. All patients demonstrated complete healing without evidence of malignant transformation. Therefore, vermilionectomy is an effective and reliable treatment for AC with severe epithelial dysplasia, providing low recurrence rates and favorable functional and aesthetic outcomes. These cases reinforce the role of vermilionectomy as the definitive treatment for high‐risk AC lesions.

## 1. Introduction

Actinic cheilitis (AC) is a prevalent potentially malignant disorder that primarily affects the lower lip as a result of prolonged exposure to ultraviolet B radiation [1, 2]. Clinically, AC is characterized by diffuse whitish plaques that may be associated with erythematous areas, erosions, or atrophy of the vermilion border. Although often asymptomatic, some patients may report dryness, discomfort, or pain. A definitive diagnosis is established through biopsy and histopathological examination, and the degree of epithelial dysplasia is a key determinant in therapeutic decision‐making [1, 2].

Treatment options include topical agents, photodynamic therapy (PDT), and surgical excision via vermilionectomy [1–3]. Regardless of the chosen modality, long‐term follow‐up is essential due to the risk of disease progression or recurrence. A systematic review and meta‐analysis by *Carneiro* et al. [4] estimated that malignant transformation occurs in approximately 14% of AC cases. Furthermore, evidence suggests that the vast majority of lower lip squamous cell carcinomas—up to 95%—originate from pre‐existing AC lesions, which may present an increased metastatic potential [3, 5].

Vermilionectomy is widely regarded as the preferred treatment approach for AC, particularly in the presence of severe epithelial dysplasia [1, 2]. The procedure consists of excising the vermilion border to the level of the underlying muscle, while preserving the muscular layer, followed by reconstruction through advancement of the labial mucosa. Although it is a more invasive technique, it is generally associated with favorable patient‐reported aesthetic outcomes and demonstrates lower rates of recurrence and malignant transformation compared with other available treatment modalities [5, 6].

Thus, this study presents three cases of AC with severe epithelial dysplasia managed with vermilionectomy, focusing on clinical and postoperative outcomes.

## 2. Case Description

### 2.1. Case 1

**Figure 1:**
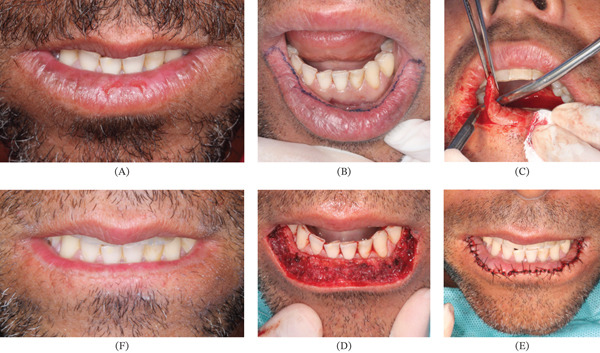
Vermilionectomy performed under local anesthesia for the treatment of actinic cheilitis. (A) Diffuse whitish plaques with erosive areas are visible on the lower lip. (B) Before anesthetic infiltration with 4% articaine containing epinephrine 1:100.000, the incision line is marked along the junction between the vermilion and the adjacent skin anteriorly, and with the labial mucosa posteriorly. (C) The incision is made using a no. Fifteen scalpel blade and electrocautery. (D) As moderate bleeding is expected, hemostasis is achieved with electrocautery by coagulating branches of the labial artery throughout the surgical field. (E) The labial mucosa is then advanced and sutured to the skin with 4‐0 nylon interrupted stitches. (F) At the 2‐year follow‐up, no clinical signs of recurrence are observed, and a satisfactory aesthetic outcome is achieved despite a notable reduction in lower lip volume.

A 41‐year‐old male patient was referred to the oral and maxillofacial surgery service following a histopathological diagnosis of AC with severe epithelial dysplasia on the lower lip. His medical history was unremarkable, and he reported no tobacco or alcohol use. However, the patient worked as a beach tennis instructor and had a history of surfing, indicating chronic sun exposure as a predisposing factor. Physical examination revealed diffuse white plaques and erosions along the lower lip vermilion (Figure 1A). Under local anesthesia and oral sedation with 15 mg of midazolam, vermilionectomy was performed. For antibiotic prophylaxis, the patient received 2 g of amoxicillin 1 h before surgery. Bilateral mental nerve block was achieved using 4% articaine with epinephrine 1:100.000, followed by local infiltration along the demarcated margins of the surgical field (Figure 1B). The incision was made using a no. Fifteen scalpel blade and electrocautery, excising the entire vermilion of the lower lip down to the muscular layer (Figure 1C). Hemostasis was achieved using a monopolar electrosurgical unit (ConMed System 5000, ConMed Corporation, United States) in coagulation mode. The device was applied in short, intermittent bursts of approximately 1–2 s, with a power setting adjusted within the standard clinical range (approximately 20–30 W), sufficient to control bleeding while minimizing thermal damage to adjacent tissues. Subsequently, the intraoral mucosa was advanced and sutured to the skin using 4‐0 nylon interrupted stitches (Figure 1D,E). The excised tissue was submitted for histopathological examination, which confirmed the absence of areas of malignant transformation. Postoperative recovery was uneventful, and sutures were removed after 10 days. During a 2‐year follow‐up period, the patient remained asymptomatic and exhibited no evidence of recurrence (Figure 1F).

### 2.2. Case 2

**Figure 2:**
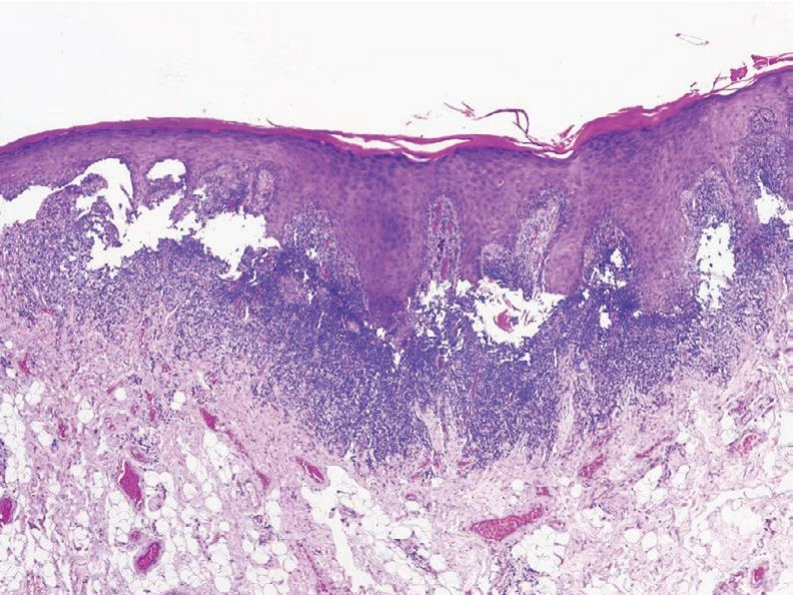
AC microscopic features: Histology shows hyperkeratosis, solar elastosis, and severe epithelial dysplasia in actinic cheilitis. (Hematoxylin and eosin stain; original magnification ×100).

**Figure 3:**
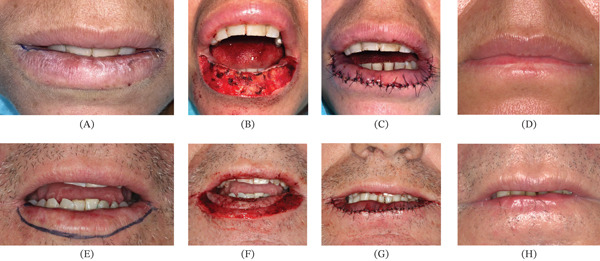
Initial clinical presentation of actinic cheilitis (AC) and postoperative outcomes following vermilionectomy of Cases 2 and 3. (A) Whitish plaques interspersed with erosive areas and atrophy of the vermilion border. At this moment, the electrocautery is used to obtain hemostasis prior to mucosal advancement. (B) Intraoperative view immediately after excision of the lower lip vermilion; electrocautery is used to achieve hemostasis before mucosal advancement. (C) Labial mucosa advanced and sutured to the skin using 4‐0 nylon interrupted stitches. (D) Postoperative aspect showing the incision scar and a marked reduction in lower lip volume, though the overall aesthetic result remains satisfactory. (E) Preoperative marking of the incision limits before local anesthetic infiltration with an anesthetic + epinephrine − containing solution. (F) Excision of the lower lip vermilion followed by blunt dissection of the labial mucosa to achieve adequate tissue mobility for tension‐free closure. (G) Multiple 4‐0 nylon interrupted sutures placed to approximate the labial mucosa and skin. (H) Postoperative view demonstrating a noticeable decrease in lower lip volume and the proximity of hair follicles to the oral mucosa, resulting from advancement of the skin during lip reconstruction.

A 45‐year‐old female patient was referred by an oral medicine specialist following an incisional biopsy that revealed AC with severe epithelial dysplasia affecting the lower lip (Figure 2). Her medical history was unremarkable, and she denied any history of tobacco or alcohol use. The patient lived in a coastal area and reported frequent sun exposure over several years without the regular use of sunscreen or protective lip balm. Clinical examination showed multiple whitish plaques interspersed with erosive areas and atrophy of the vermilion border (Figure 3A). Therefore, vermilionectomy was proposed and performed under local anesthesia (Figure 3B,C). The excised specimen was submitted for histopathological reassessment, which confirmed the previous diagnosis of AC with severe epithelial dysplasia. Postoperative recovery was uneventful, and sutures were removed after 10 days. At the 1‐year follow‐up, the patient showed no symptoms and no clinical signs of recurrence (Figure 3D).

### 2.3. Case 3

A 53‐year‐old male was referred by a general dentist for evaluation of whitish lesions on the lower lip. His medical history was otherwise unremarkable, although he reported chronic tobacco and alcohol use. Clinical examination revealed diffuse white plaques accompanied by atrophy of the vermilion border and erosive areas (Figure 3E). Based on the clinical findings, a provisional diagnosis of AC was established, and an incisional biopsy was performed under local anesthesia. Histopathological evaluation confirmed AC with severe epithelial dysplasia. One month later, the patient underwent vermilionectomy under local anesthesia combined with oral sedation (Figure 3F,G). The excised tissue was sent for microscopic evaluation, which showed no evidence of malignant transformation. On the fifth postoperative day, partial wound dehiscence was observed in localized areas and managed conservatively with topical antibiotic ointment. Sutures were removed on Day 10, and healing progressed satisfactorily. At the 1‐year follow‐up, the patient demonstrated full recovery, with no evidence of recurrence either clinically or symptomatically (Figure 3H).

## 3. Discussion

Vermilionectomy with subsequent lip reconstruction was first described by *von Langenbeck–von Bruns* in 1857 [7] and has since been established as an effective surgical technique for the management of precancerous lesions of the lower lip, such as AC. The procedure provides both functional restoration and satisfactory aesthetic outcomes. Several studies have reported minimal to no recurrence of AC following vermilionectomy [8, 9], and the technique is also widely used in the management of lower lip squamous cell carcinoma [5]. In the present series, all patients demonstrated complete healing, with no evidence of recurrence during a minimum follow‐up period of 1 year.

Microscopically, AC may present with varying degrees of epithelial dysplasia—mild, moderate, or severe—with the latter associated with the highest risk of malignant transformation [1–4]. Therefore, the degree of dysplasia plays a critical role in guiding therapeutic decisions. Patients with mild to moderate dysplasia may be managed with less invasive approaches, including topical agents or PDT [2, 10]. These modalities generally provide satisfactory clinical and histological outcomes and are associated with minimal morbidity [5]. However, they tend to present higher recurrence rates and may be insufficient in cases of severe epithelial dysplasia.

In the management of AC, different treatment modalities present distinct advantages and limitations. Vermilionectomy enables complete removal of the affected epithelium, providing both definitive treatment and full histopathological evaluation of the lesion [5, 6]. In contrast, PDT is a minimally invasive approach associated with favorable cosmetic outcomes [2]; however, it often requires multiple treatment sessions and demonstrates variable efficacy, particularly in cases of severe epithelial dysplasia. Moreover, PDT does not allow comprehensive histopathological assessment, which may increase the risk of residual disease [2, 6]. Similarly, laser‐based techniques, such as CO_2_ and Er:YAG laser vaporization, allow precise ablation of affected tissues with reduced intraoperative bleeding and postoperative discomfort [9]. Nevertheless, like PDT, these approaches do not permit complete histopathological evaluation and may result in incomplete removal of dysplastic areas [2]. Therefore, although less invasive approaches may be appropriate for selected low‐risk cases, vermilionectomy continues to represent the most dependable and definitive option for high‐risk lesions—especially those with severe epithelial dysplasia—due to its lower recurrence rates and greater diagnostic reliability [2–10].

Recurrence rates after vermilionectomy are consistently reported as low in the literature, generally ranging from 0% to 5%, depending on follow‐up duration and study design [8, 9]. In contrast, nonsurgical approaches such as PDT have demonstrated higher recurrence rates, varying between approximately 10% and 30% in different clinical series [2, 8, 10]. Moreover, laser‐based techniques, such as CO_2_ and Er:YAG laser vaporization, have shown intermediate recurrence rates, typically ranging from approximately 5% to 21%, depending on lesion characteristics and follow‐up duration [8, 9]. Therefore, these findings reinforce the role of vermilionectomy as the definitive treatment modality, particularly for lesions with severe epithelial dysplasia.

Vermilionectomy can be safely performed under local anesthesia with or without sedation, although general anesthesia may be indicated in selected cases [5]. Regardless of the anesthetic approach, effective intraoperative hemostasis is essential, as significant bleeding is expected due to the rich vascularization of the lip. Electrocautery and surgical lasers are commonly used for this purpose. Potential complications include hematoma, wound dehiscence, visible scarring, reduction in lip volume, and paresthesia [5, 11]. Reconstruction through advancement of the labial mucosa typically results in an approximate 30% reduction in lower lip volume; however, the use of grafting techniques may help minimize this outcome [11, 12].

In the present study, all patients underwent vermilionectomy under local anesthesia and achieved satisfactory clinical outcomes. Complete healing was observed in all cases, with no recurrence during follow‐up. Additionally, the assessment of aesthetic outcomes and patient satisfaction was based on clinical observation during follow‐up visits rather than on standardized objective measurement tools or validated questionnaires. In all cases, aesthetic outcomes were considered satisfactory by both the clinician and the patients, based on lip contour, color match, and functional recovery, including speech and oral competence. This constitutes a limitation of the present study; therefore, future research should include validated tools to provide objective evaluation of aesthetic outcomes and patient satisfaction following vermilionectomy.

## 4. Conclusion

Vermilionectomy remains a reliable and effective treatment for AC, particularly in cases of severe epithelial dysplasia, where noninvasive therapies may be insufficient. The procedure offers both functional restoration and satisfactory aesthetic outcomes, with a low risk of recurrence when performed correctly.

## Author Contributions

Bruno Teixeira Gonçalves Rodrigues: conceptualization, formal analysis, and writing—original draft. Eduardo Martins Falcão, Allan Ribeiro Bastos, Verônica dos Santos Lopes Serrano, Caroline Cavaignac Silva, Ana Carolina Silva Custódio, and Paula Granha Porto: writing—review and editing. Danilo Passeado Branco Ribeiro, Henrique Martins da Silveira, and Mônica Simões Israel: conceptualization, formal analysis, and writing—review and editing.

## Funding

This study was financed in part by the CoordenaÃ§Ã£o de AperfeiÃ§oamento de Pessoal de NÃvel Superiorâ€ “Brasil (CAPES)â€”Finance Code 001.

## Disclosure

All authors have read and approved the final version of the manuscript. Mônica Simões Israel had full access to all of the data in this study and takes complete responsibility for the integrity of the data and the accuracy of the data analysis.

## Ethics Statement

Data from the patients here included were treated anonymously and a statement of informed consent was signed by the patients allowing the use of their dental records.

## Conflicts of Interest

The authors declare no conflicts of interest.

## Data Availability

Data sharing is not applicable to this article as no datasets were generated or analyzed during the current study.
